# Thyroid and Pregnancy

**DOI:** 10.4061/2012/680328

**Published:** 2011-11-16

**Authors:** Bijay Vaidya, Roberto Negro, Kris Poppe, Joanne Rovet

**Affiliations:** ^1^Department of Endocrinology, Royal Devon & Exeter Hospital and Peninsula Medical School, Exeter EX2 5DW, UK; ^2^Division of Endocrinology, V. Fazzi Hospital, Lecce, Italy; ^3^Department of Endocrinology, UZ Brussel VUB, Brussels, Belgium; ^4^Department of Paediatrics, The Hospital for Sick Children, Toronto, ON, Canada

In the last two decades, there have been major advances in our understanding of the thyroid physiology during pregnancy, the role of thyroid hormones in fetal development, and the effects of thyroid dysfunction on pregnancy outcomes. The main objective of this special issue was to highlight how these advances have enhanced our knowledge and influenced the clinical practice in the field.

The emerging evidence for an association between thyroid autoimmunity and spontaneous miscarriages is one of such advances. As A. Stagnaro-Green reviews in this special issue, since the publication of the first report describing the association in 1990 [1], many subsequent studies have lent further evidence to support this association. However, despite the robust evidence for the association, the pathogenesis of miscarriages in pregnant women with thyroid autoimmunity remains uncertain, and whether levothyroxine treatment prevents the adverse outcome in these women is yet to be confirmed.

For many decades, it has been recognised that overt maternal hypothyroidism during pregnancy is associated with impaired neurological development of the offspring; however, several studies in the recent years have suggested that even mild maternal thyroid hormone deficiency (subclinical hypothyroidism and isolated maternal hypothyroxinaemia) during pregnancy can affect the offspring's neuropsychological development [2, 3]. However, this association has not been consistent in all studies [4], and J. Chevrier and colleagues in this special issue report a prospective study showing lack of association between maternal thyroid hormone levels at 27-week gestation and neuropsychological development of the offspring. Indeed, as M. Moleti and colleagues highlight in their review, there is also a great deal of controversy surrounding the diagnosis, adverse effects, and management of isolated maternal hypothyroxinaemia in pregnancy, and more studies are needed to resolve these controversies.

The last two decades have also witnessed significant advances in the diagnosis and management of hypothyroidism in pregnancy. The importance of trimester-specific reference ranges for thyroid function tests in pregnancy has been established [5, 6]; it has become clear that the upper reference limit of serum thyrotropin (TSH) in pregnancy is much lower than that in the general population. It has also been convincingly shown that most hypothyroid women need an increased dose of levothyroxine during pregnancy. However, there remains uncertainty at what level of TSH should the levothyroxine replacement be considered and whether women with isolated maternal hypothyroxinaemia or isolated positive thyroid peroxidase antibodies should be treated with levothyroxine. Furthermore, there is no consensus on whether all pregnant women should be screened for hypothyroidism. J. Klubo-Gwiezdzinska and colleagues review the issues surrounding indications, efficacy, and monitoring of levothyroxine replacement in pregnancy, and J. H. Lazarus appraises evidences for and against screening all pregnant women for thyroid dysfunction. The current guidelines from the Endocrine Society and the American Thyroid Association do not endorse universal screening of pregnant women for thyroid dysfunction but recommend case-finding approach in high-risk pregnant women [5, 6]. However, V. Nambiar and colleagues show, in the Asian-Indian population, that the case-finding approach misses a significant proportion of pregnant women with thyroid dysfunction, in line with findings of several studies from the western countries. Their study also provides further evidence to support that both maternal thyroid autoimmunity and maternal mild hypothyroidism are associated with an increased risk of spontaneous miscarriages. And, although not as fiercely debated as screening pregnant women for hypothyroidism, there is also lack of consensus on screening for postpartum thyroiditis. M. A. Adlan and L. D. Premawardhana review the issues surrounding screening for postpartum thyroiditis and the utility of thyroid peroxidase antibodies testing as a screening tool for this condition. 

In pregnancy, Graves' disease is the commonest cause of hyperthyroidism, and thionamide antithyroid drugs are the mainstay of treatment for this condition. However, in the recent years, reports of rare association of carbimazole (and its active metabolite, methimazole) use in early pregnancy with multiple congenital malformations in the foetus and association of propylthiouracil with severe liver injury have led to the controversy surrounding the choice of antithyroid drugs in pregnancy. For example, which antithyroid drug should be prescribed for a woman with Graves' disease planning pregnancy? If a pregnant woman is on propylthiouracil, should the drug be switched to carbimazole (or methimazole) after the first trimester? In this special issue, P. Bowman and B. Vaidya, by analysing all birth defects related to maternal treatment of carbimazole and propylthiouracil reported to the UK Pharmacovigilance authority over a 47-year period, provide further evidence to support an embryopathy associated with carbimazole exposure in utero. However, their study also raises a question whether the currently apparent lack of association of similar embryopathy with propylthiouracil is related to historically lower use of the drug as compared to carbimazole or methimazole. Furthermore, transient gestational hyperthyroidism—another common cause of hyperthyroidism in pregnancy—is often confused with Graves' disease, sometimes leading to inappropriate treatment. A. M. Goldman and J. H. Mestman review the aetiology, pathogenesis, diagnosis, and management of this intriguing condition.

The association between severe iodine deficiency and cretinism has been known for more than a century [7]. Furthermore, recent studies have shown that mild iodine deficiency is also associated with impaired cognitive and behaviour outcomes in the children, including attention deficit hyperactivity disorder. Despite these observations and all national and international efforts to optimise dietary iodine intake in the population, iodine deficiency during pregnancy continues to be a major preventable cause of mental retardation in many countries. In fact, recent studies suggest that iodine deficiency is on the rise in Europe, Australia, and the USA [8–10]. C. Yarrington and E. N. Pearce review the adverse effects of dietary iodine deficiency on maternal thyroid function and fetal neurological outcomes and discuss the recommendations for optimum dietary iodine intake during pregnancy. 

As a byproduct of a modern life, humans are increasingly being exposed to environmental endocrine disrupting chemicals, with potential harmful health consequences. Recent studies suggest that some of these chemicals could interfere with normal thyroid hormone function. Therefore, there is a growing concern that an exposure to these chemicals during pregnancy may adversely affect maternal and fetal thyroid function impacting on the fetal development, as M.-L. Hartoft-Nielsen and colleagues discuss in their review.

Finally, although fortunately rare, thyroid cancer presents special challenges in the management during pregnancy. S. A. Imran and M. Rajaraman discuss various issues surrounding management of differentiated thyroid cancer in pregnancy and underline the importance of multidisciplinary approach in the management. However, much of the clinical decisions in the management of thyroid cancer in pregnancy are hampered by the lack of good quality evidence, as G. V. Alves and colleagues highlight in their systemic review.

We believe that the papers in this special issue illustrate the highlights of advances made in the diverse areas of thyroid and pregnancy over the last two decades. At the same time, they also underline many yet unanswered questions and areas for further studies. However, with the volume and the quality of ongoing research activities in the field, we are optimistic that we will not need to wait for a further two decades to have the answers for many of these questions. 

*Bijay Vaidya* *Bijay Vaidya* *Roberto Negro* *Roberto Negro* *Kris Poppe* *Kris Poppe* *Joanne Rovet* *Joanne Rovet*
